# Enhanced Solubility and Antitumor Activity of Annona Squamosa Seed Oil via Nanoparticles Stabilized with TPGS: Preparation and In Vitro and In Vivo Evaluation

**DOI:** 10.3390/pharmaceutics14061232

**Published:** 2022-06-10

**Authors:** Hui Ao, Likang Lu, Manzhen Li, Meihua Han, Yifei Guo, Xiangtao Wang

**Affiliations:** Institute of Medicinal Plant Development, Chinese Academy of Medical Sciences & Peking Union Medical College, No. 151, Malianwa North Road, Haidian District, Beijing 100193, China; aohui@implad.ac.cn (H.A.); lulikang@implad.ac.cn (L.L.); limanzhen@implad.ac.cn (M.L.); mhhan@implad.ac.cn (M.H.); yfguo@implad.ac.cn (Y.G.)

**Keywords:** annona squamosa seed oil, nanoparticles, stability, antitumor activity, tumor targeting

## Abstract

Annona squamosa seed oil (ASSO), which is a waste product in the extraction of annonaceous acetogenins (ACGs), displays good antitumor activity against a variety of tumor cells. However, ASSO is insoluble and has low bioavailability. In order to improve the solubility and application value of ASSO, the seed oil nanoparticles (ASSO-NPs) were successfully prepared only using TPGS as a stabilizer. ASSO-NPs obtained were spherical with a uniform size (less than 200 nm). ASSO-NPs showed the good storage stability at 25 ± 2 °C and were suitable for both oral administration and intravenous injection. The antitumor study in vitro and in vivo demonstrated more enhanced antitumor efficacy of ASSO-NPs than free ASSO. The ASSO-NPs group (15 mg/kg) had the highest tumor inhibition rate (TIR) of 69.8%, greater than the ASSO solution (52.7%, 135 mg/kg, *p* < 0.05) in 4T1 tumor-bearing mice. The in vivo biodistribution data displayed that the fluorescence intensity of ASSO/DiR-NPs in tumor was similar to that in liver in the presence of the reticuloendothelial system. Besides, the relative tumor-targeting index (RTTI) of (ACGs + ASSO)-NPs was 1.47-fold that of ACGs delivered alone, and there is great potential in ASSO-NPs as tumor-targeted delivery vehicles. In this study, ASSO-NPs were firstly prepared by a very simple method with fewer excipients, which improved the solubility and antitumor activity of the ASSO, displaying a good prospect in the in vivo delivery of natural bioactive compounds.

## 1. Introduction

The Annonaceae family plants are native to America and mainly distributed in the tropics, which are composed of approximately 135 genera and 2300 species in the world [1,2,3]. Plants in the Annonaceae family contain alkaloids, terpenes, flavonoids, annonaceous acetogenins, polyoxycyclohexene, and styrolactone, among which annonaceous acetogenins is widely concerned for its strong antitumor activity [4]. There are many medicinal values in Annona squamosa plant that belongs to the genus Annona in the Annonaceae family. The ingredients in Annona squamosa bark have an anti-ulcer effect, as well as analgesic and anti-inflammatory activity [5,6]. Annona squamosa leaves have anti-fungal and anti-oxidant activities and antitumor efficacy [7,8,9]. In particular, the Annona squamosa seeds have strong antitumor activity due to the presence of annonaceous acetogenins (ACGs) [10,11,12].

Annonaceous acetogenins are mainly extracted from seeds of Annonaceae plants [13], and ASSO is separated during the extraction process, which is generally discarded. However, it has been reported that Annona squamosa seed oil (ASSO) has anti-oxidant activity [14], anti-psoriatic activity [15], lipoxygenase inhibitory activity [16], and antitumor activity. Yong Chen et al. [17] established a H22 tumor-bearing mice model, proving that the TIR of ASSO at the dose of 1.0 mL/kg was 53.54%. Yan Qiu et al. [18] investigated the cell proliferation-inhibitory activity of ASSO by MTT assay, indicating the half inhibitory concentration (IC50) against SMMC-7721, HepG2, HeLa, A-549, and MCF-7 cells, which were 10.4, 0.57, 30.3, 44.3, and 6.7 mg/L, respectively. In order to improve the use value of Annona squamosa seeds, antitumor studies were carried out using ASSO as a model drug.

**Scheme 1:**
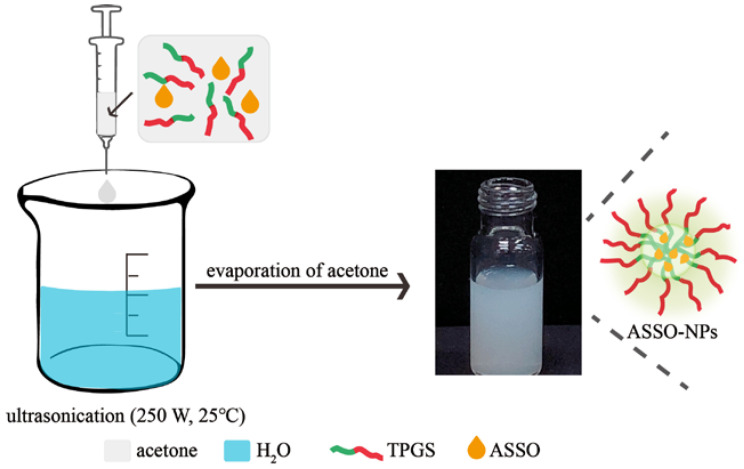
The schematic illustration of the preparation for ASSO-NPs with the best method.

Obviously, ASSO is insoluble in water, which results in the low bioavailability [19] and greatly limits its wide use. In addition, chemotherapeutic drugs often have serious side effects because they have no selective killing effect on normal cells and tumor cells [20]. Inclusion complex [21], micelle [22], nanoemulsion [23], or microemulsion [24] are commonly used to increase the solubility of oil, but more excipients are needed to help it exist stably and the drug loading content is low. In order to improve the solubility, bioavailability, and passive targeting on tumor cells of ASSO, nanotechnology was used [25]. In this study, only TPGS was used as a stabilizer to prepare Annona squamosa seed oil nanoparticles (ASSO-NPs) successfully by ultrasonic-dripping method [26], which was reported for the first time (Scheme 1). It was kept at room temperature for 45 days without significant change in particle size and appearance. The antitumor study in vitro and in vivo demonstrated more enhanced antitumor efficacy of ASSO-NPs than free ASSO. Interestingly, ASSO-NPs had great potential as tumor-targeted delivery vehicles. In vivo distribution data showed that the relative tumor-targeting index (RTTI) of (ACGs + ASSO)-NPs was 1.47-fold that of ACGs delivered alone. 

## 2. Materials and Methods

### 2.1. Materials

ASSO was extracted from Annona squamosa seeds by the Institute of Medicinal Plant Development (IMPLAD, Beijing, China). ACGs were provided by Professor Jianyong Si’s laboratory (IMPLAD, Beijing, China). d-α-tocopherol polyethylene glycol 1000 succinate (TPGS) was purchased from Xi’an Healthful Biotechnology Co. Ltd. (Xi’an, China). Sodium oleate was obtained from BioRuler (Danbury, CT, USA), Solutol^®^ HS 15 was purchased from BASF SE (ludwigshafen, Germany), dodecyl sodium sulfate (SDS) was provided by Sinopharm Chemical Reagent Co., Ltd. (Shanghai, China), paclitaxel (PTX) injections were obtained from Beijing Union Pharm Ltd. (Beijing, China), and all the other reagents were of analytical grade or higher. In addition, deionized water was used.

### 2.2. Cell Lines and Animals 

Mouse breast cancer cells (4T1) were purchased from China’s infrastructure of cell line resource and cultured in Roswell Park Memorial Institute 1640 medium (RPMI 1640, HyClone, Waltham, MA, USA) supplemented with 10% fetal calf serum (FBS) and 100 U/mL penicillin-streptomycin (Gibco, St Louis, MO, USA) at 37 °C with 5% CO_2_ (Thermo311, Waltham, MA, USA).

Thirty-three female BAL B/c mice (20 ± 2 g, 6–8 weeks old) were purchased from Vital River Laboratory Animal Technology Co., Ltd. (Beijing, China). All mice were kept under SPF environments and all animal experiments were conducted on the basis of the Guidelines for Ethical and Regulatory for Animal Experiments as defined by Institute of Medicinal Plant Development (IMPLAD), China. The ethics committee of IMPLAD granted ethical approval for this study and the approval number of the ethics committee was SLXD-20200827014.

### 2.3. Extraction of ASSO

The ASSO was obtained by ultrasonic extraction method. Simply, the mixture of Annona squamosa seed powder and petroleum ether (*w*/*v*, 1/10) was sonicated for 1 h (250 W, 25 °C) and then filtered. The seed oil can be acquired by evaporation of petroleum ether at 45 °C under reduced pressure.

### 2.4. Preparation of ASSO-NPs

#### 2.4.1. Screening Suitable Stabilizer of ASSO-NPs

Sodium oleate, Solutol^®^ HS 15, SDS, and TPGS were chosen to prepare ASSO-NPs by ultrasonic-dripping method. Ten milligrams of TPGS and 20 mg ASSO were dissolved in 0.5 mL acetone as organic phase, and they were slowly dropped into 5 mL of deionized water at 25 ± 2 °C under the condition of 250 W ultrasonication (Kun Shan Ultrasonic Instruments Co., Ltd., Kunshan, China); or only 20 mg ASSO was dissolved in acetone as the organic phase and dropped into 5 mL deionized water with 10 mg stabilizer (sodium oleate, Solutol^®^ HS 15 or SDS) dissolved. The ASSO-NPs (4 mg/mL) were obtained by the evaporation of acetone at 40 °C. The best stabilizer was selected according to the particle size and polydispersity index (PDI) of ASSO-NPs.

#### 2.4.2. Optimizing ASSO/Stabilizer Ratio and Preparation Concentration of ASSO-NPs

The ASSO/stabilizer ratios of 2:1, 1:1, and 1:2 and the preparation concentrations of 2, 4, and 6 mg/mL were screened to attain ASSO-NPs with smaller particle size. The operations were as described above. Based on the particle size and PDI of ASSO-NPs, the optimal ASSO/ stabilizer ratio and preparation concentration were chosen.

### 2.5. Characterization of ASSO-NPs

#### 2.5.1. Dynamic Light Scattering Measurement

Dynamic light scattering instrument (DLS; Zetasizer Nano ZS, Malvern Instruments, Malvern, UK) with a He–Ne laser (633 nm) was used to detect the mean particle size, PDI, and zeta potential of ASSO-NPs at 25 °C. ASSO-NPs were diluted to 1.0 mg/mL with deionized water prior to DLS analysis. One milliliter of the diluted sample was placed in a disposable sizing cuvette to determine the average particle size and PDI of ASSO-NPs at a scattering angle of 173°; 0.4 mL of the sample was placed in a disposable folded capillary zeta cell for zeta potential determination at a scattering angle of 13°, and the water was used as a dispersant with a viscosity (0.89 cP), refractive index (RI, 1.33), and dielectric constant (78.5). The zeta potential can be converted based on Henry function (1). Each measurement was performed in triplicate and with 12 runs.

ζ = 3ηU_E_/2εf(Ka) (1)

ζ: zeta potential; η: viscosity of the dispersant; U_E_: electrophoretic mobility; and ε: dielectric constant and f(Ka):1.5.

#### 2.5.2. Morphology of ASSO-NPs

The morphology of ASSO-NPs was observed using a JEM-1400 transmission electron microscope (TEM; JEOL, Tokyo, Japan). Briefly, ASSO-NPs were dropped on the surface of a 300-mesh copper mesh, stained with 2% (*w/v*) uranyl acetate after air drying, and observed under an electron microscope.

#### 2.5.3. FT-IR Analysis

The ASSO, TPGS, physical mixtures of ASSO and TPGS (mass ratio of 1:2), and the lyophilized powder of ASSO-NPs were placed on the sample table and compressed by ATR probe, respectively. 

In particular, ASSO and TPGS were heated to 50 °C, then fully mixed under sonication to prepare their physical mixture. The samples were scanned by the Fourier transform-infrared spectroscopy (FT-IR, Spectrum Two, PerkinElmer, USA) in the range of 4000–500 cm^−1^ with a resolution of 2 cm^−1^ and scanning eight times at 25 °C to obtain the FT-IR spectra.

### 2.6. The Storage Stability and the Stability of ASSO-NPs in Physiological Media

The storage stability of ASSO-NPs was determined by placing the ASSO-NPs at 25 ± 2 °C and measuring particle size at specific time points to observe the size change during storage. The stability of ASSO-NPs in physiological media was investigated by mixing ASSO-NPs with NaCl solution (1.8%), glucose solution (10%), and 2× PBS (pH 7.4) (1:1, *v*/*v*), or mixing with artificial gastric juice, artificial intestinal fluid, and plasma (1:4, *v*/*v*), respectively. Then these mixtures were incubated at 37 °C. The particle size of these mixtures was measured by DLS at the set time. The above experiments were conducted in triplicates.

### 2.7. The Pharmacological Evaluation of ASSO-NPs

#### 2.7.1. In Vitro Cytotoxicity Assay

The cytotoxicity of ASSO solution and ASSO-NPs was researched by the MTS cell proliferation experiments. 4T1 cells in logarithmic phase were seeded in 96-well plates (5000 cells/per) and cultured at 37 °C with 5% CO_2_ for 24 h. Then, the complete media was replaced by ASSO solutions with 0.5% DMSO as the negative control group or ASSO-NPs with complete media as the negative control group for an additional 72 h. Cell viability was assessed with MTS assay kit (MTS, Promega, Madison, WI, USA). Briefly, 20 μL of Cell Titer 96^®^ Aqueous One Solution was added to each well and incubated with cells at 37 °C for 3 h. Finally, absorbance was detected at 490 nm using a plate reader (Synergy H1, Biotek, Winooski, VT, USA). The cell viability rate was calculated as Equation (2):

Cell viability rate (%) = (ODe/ODc) × 100%(2)

In which ODe and ODc are respectively the mean optical density of the experimental group and control group.

The half inhibitory concentration (IC50) value was calculated using GraphPad Prism Software, Version 5 (GraphPad Software, Inc., La Jolla, CA, USA).

#### 2.7.2. In Vivo Antitumor Activity in 4T1 Tumor-Bearing Mice

4T1 cells in logarithmic phase were suspended in RPMI medium without FBS, and 0.2 mL cell suspensions (1 × 10^7^ cells/mL) were inoculated subcutaneously into the right armpit of female BAL B/c mice. The mice were fed in the SPF environment, and the growth of the tumor was closely watched. When the tumor size reached about 100 mm^3^, the mice were randomly divided into four groups (*n* = 6) as follows: normal saline (as negative control), PTX injection (10 mg/kg, iv, as positive control), ASSO solution (135 mg/kg, ig), and ASSO-NPs (15 mg/kg, iv). The negative control group and the positive control group were injected via the lateral tail vein every other day 7 times. The ASSO solution group was orally administered every day 5 times, stopped for 2 days, and then given every other day 3 times. The ASSO-NPs group was given intravenously every other day 3 times, stopped for 2 days, and then given every other day 3 times. The volume of tumors and the mice weight were measured every 2 days during the experiment period. The mice were sacrificed by cervical vertebra dislocation and dissected 24 h after the last dose. The TIR was calculated as Equation (3), and the liver index or spleen index was calculated as Equation (4).

TIR (%) = (1 − We/Wn) × 100%(3)

In which We and Wn are respectively the mean tumor weight of the experimental group and negative control group.

Liver index or spleen index = W_1_/W_2_ (4)

In which W_1_ is the mean liver or spleen weight of the experimental group, and W_2_ is the mean mice weight of the negative control group.

### 2.8. In Vivo Biodistribution Study Based ASSO-NPs

DiR, a lipophilic fluorescent dye, can exhibit a specific color at a specific wavelength. In order to visually observe the distribution of ASSO-NPs in 4T1 tumor-bearing mice, ASSO-NPs-loaded DiR was prepared as the method “Preparation of ASSO-NPs”, except that ASSO was replaced by a mixture of ASSO and DiR (ASSO:DiR = 250: 1, *w*/*w*). 4T1 tumor-bearing mice model was established as described above. When the tumor size reached about 500 mm^3^, tumor-bearing mice were injected intravenously with ASSO/DiR-NPs (10 mg/kg) and were imaged using IVIS Living Image^@^ 4.4 (Caliper Life Sciences, Hopkinton, MA, USA) at a set time after administration. After 24 h, the mice were sacrificed, and the tumors and major organs were excised, then they were imaged as described. Living Image software (version 4.2) was used for quantitative analysis. The relative tumor-targeting index (RTTI) was calculated according to the following Equation (5):

(5) $$\mathrm{RTTI}=\frac{{\mathrm{ROI}}_{t}}{{\mathrm{ROI}}_{l}}$$

In which ROI_t_ and ROI_l_ are respectively the average fluorescence intensity of tumor and liver.

In addition, to investigate the feasibility of ASSO-NPs as tumor-targeted delivery vehicles, ASSO-NPs were loaded with ACGs to obtain (ACGs + ASSO)-NPs, and ACGs-NPs were prepared without ASSO, according to the method reported in the literature [27]. Both nanoparticles were labelled with DiR. Forty-eight hours after administration, the mice were sacrificed, and the tumors and major organs were excised, then they were imaged and the RTTI was calculated based on Equation (5). 

### 2.9. Statistical Analysis

The statistical analysis among the different groups was conducted using IBM SPSS Statistics software, Version 19 (IBM Corporation, Armonk, NY, USA). *p* < 0.05 was considered statistically significant.

## 3. Results and Discussion

### 3.1. Extraction of ASSO

The extraction process of the ASSO was simple and had a high yield of 24%. The density of the ASSO measured by specific gravity method was approximately 0.91 g/mL. The chemical composition of the ASSO has been analyzed in a lot of literature and was mainly composed of oleic acid, linoleic acid, stearic acid, and palmitic acid, of which unsaturated fatty acids account for about 70% [14,15,17,18].

### 3.2. Preparation of ASSO-NPs

#### 3.2.1. Screening Suitable Stabilizer of ASSO-NPs

The stabilizer plays a very important role in the nano drug delivery system, which can not only improve the stability of the nanoparticles but also control the release of the drug, improve the bioavailability of the drug, and change the in vivo distribution and circulation time of the drug [28,29,30,31]. In this study, the stabilizers for the preparation of ASSO-NPs were screened based on small particle size and narrow particle size distribution. As shown in Table 1, when Solutol^®^ HS 15 or SDS were used as the stabilizer, the average particle size of the ASSO-NPs was greater than 300 nm, and the average PDI value was greater than 0.3. While, when TPGS was used as the stabilizer, the particle size of ASSO-NPs was 253.1 ± 7.6 nm and the PDI value was 0.28 ± 0.03, showing a smaller particle size and a narrower particle size distribution. Therefore, TPGS was selected as a stabilizer to prepare ASSO-NPs. 

As seen in Table 1, at the ASSO/stabilizer ratio of 2:1, the resultant ASSO-NPs containing 4 mg/mL equivalent of ASSO showed a mean particle size of 253.1 nm, which was a little larger than that needed for biomedical application. High-pressure homogenization technique [32,33], the most widely used method to decrease the particle size of nanoparticles, was used to try to reduce its particle size, but with very limited improvement (only about 30 nm reduced). Therefore, decreasing the ASSO/stabilizer ratio and/or the final equivalent ASSO concentration was used as an alternative strategy to reduce the particle size.

#### 3.2.2. Optimizing Prescription of ASSO-NPs

First, the preparation concentration was set to 4 mg/mL and the ASSO/stabilizer ratio (2:1, 1:1, 1:2) was changed with TPGS as a stabilizer. The data is shown in Table 2. When the ASSO/stabilizer ratio was 2:1 or 1:1, the mean particle size of ASSO-NPs was greater than 250 nm. while the ASSO/stabilizer ratio was 1:2, the smaller particle size with 209.8 ± 2.1 nm was shown. Therefore, the drug/stabilizer ratio of 1:2 was chosen. Then, the drug/stabilizer ratio was set to 1:2 and the preparation concentration was changed. The results showed that ASSO-NPs had the smallest particle size of 193.7 ± 4.1 nm and the narrowest particle size distribution (PDI of 0.10 ± 0.09) with the concentration of 2 mg/mL. Undoubtedly, the formulation of ASSO-NPs was determined as: the drug/stabilizer ratio was 1:2, and the optimal preparation concentration was 2 mg/mL with TPGS as a stabilizer.

### 3.3. Characterization of ASSO-NPs

**Figure 1:**
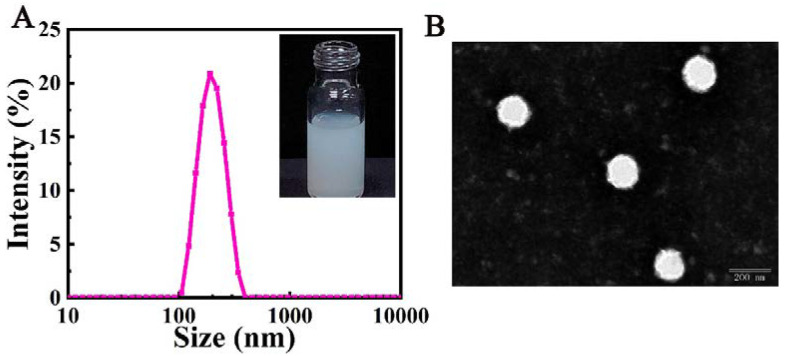
Characterization of ASSO-NPs. (**A**) Size distribution and appearance of ASSO-NPs. (**B**) Morphology of ASSO-NPs.

The dynamic light-scattering assay showed that ASSO-NPs obtained with the best prescription had a particle size of 193.7 ± 4.1 nm and a small PDI value of 0.10 ± 0.09. Figure 1A showed the particle size distribution of ASSO-NPs and their milk-like appearance. Under the electron microscope, ASSO-NPs were spherical with a uniform size, and the particle size was about 150 nm (Figure 1B). The difference in particle size between the two measurement methods was mainly because the particle size was measured in a water environment by dynamic light scattering method in which the PEG chain on the surface of ASSO-NPs was fully stretched in water, and the drying process was required when observing the morphology of ASSO-NPs under an electron microscope, which resulted in the particle size obtained by the dynamic light scattering method being larger than that measured under an electron microscope.

**Figure 2:**
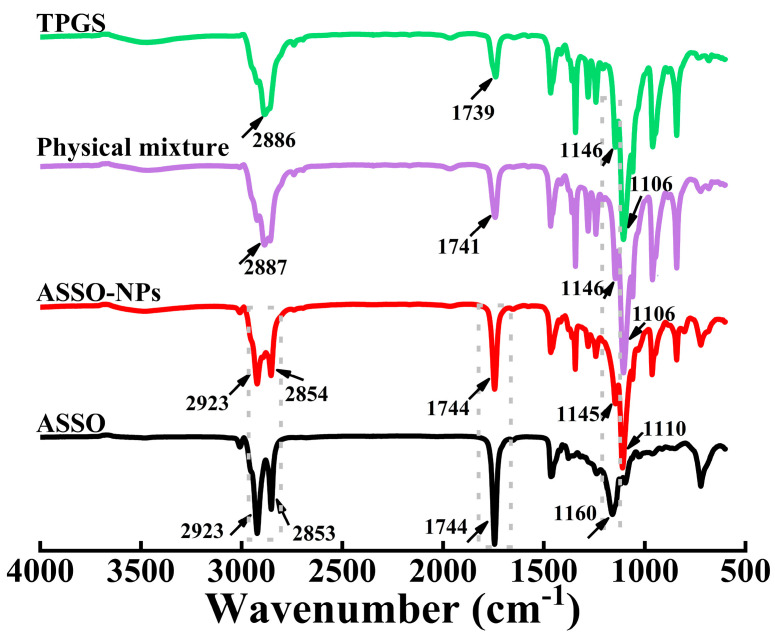
The FT-IR spectra of the ASSO, TPGS, and ASSO-NPs, and the physical mixture of ASSO and TPGS.

As described above, the ASSO was mainly composed of unsaturated fatty acids, such as oleic acid and linoleic acid. FT-IR was used here to investigate the interaction between ASSO and TPGS in ASSO-NPs. In the spectrum of ASSO (Figure 2), the sharp and strong peaks at 2923 cm^−1^ and 2853 cm^−1^ were associated with the O-H and saturated C-H stretching vibration in ASSO. The C=O stretching vibration peak was at 1744 cm^−1^, and the C-O stretching vibration peak was at 1160 cm^−1^. 

It is noted that the physical mixture and TPGS displayed very a similar FT-IR spectrum. This may be because in the physical mixture, ASSO and TPGS were nearly in the form of molecular solution, so that the -COOH groups of unsaturated fatty acids in ASSO could easily form hydrogen bonds with the oxygen atoms of the PEG segment in TPGS. Since there are about 22 oxygen atoms in the PEG segment in a single TPGS molecule, and the mass ratio of TPGS/ASSO was 2:1, nearly all the -COOH groups of the fatty acids in ASSO formed a hydrogen bond in the physical mixture, resulting in the disappearance of sharp peaks at 2923 cm^−1^ and 2853 cm^−1^, which were characteristic absorption peaks of the ASSO. 

In the core-shell structure of ASSO-NPs, the ASSO was mainly located in the core, while TPGS was mainly in the shell with the hydrophilic PEG segment fully extending into the aqueous phase. This resulted in the segregation of ASSO from the PEG segment of TPGS, thus no hydrogen bond formed for the -COOH groups of ASSO, and the sharp and strong peaks at 2923 cm^−1^ and 2854 cm^−1^ were related to the O-H and saturated C-H stretching vibration of fatty acids in ASSO, which are displayed in the FT-IR spectrum for ASSO-NPs.

Besides, there was a hydrophobic interaction between ASSO and TPGS, but it was difficult to distinguish in the FT-IR spectra as the hydrophobic interaction exited both in the physical mixture and in ASSO-NPs.

### 3.4. The Storage Stability and the Stability of ASSO-NPs in Physiological Media

**Figure 3:**
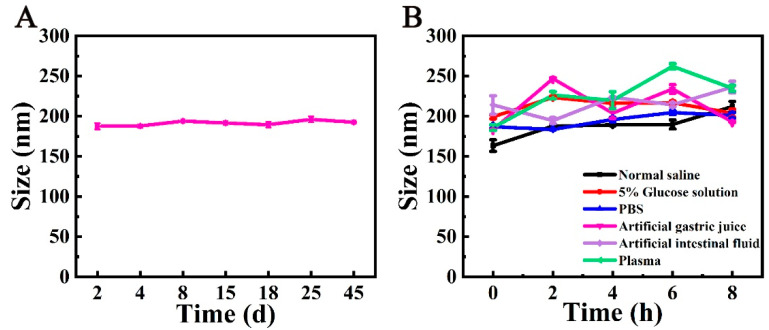
The storage stability and the stability of ASSO-NPs in physiological media. (**A**) The particle size change curve of ASSO-NPs at 25 ± 2 °C. (**B**) The particle size change curve of ASSO-NPs in physiological media.

The storage stability of a drug is very important for its transportation, preservation, and clinical use, so the storage stability of ASSO-NPs was investigated. As shown in Figure 3A, when the ASSO-NPs were placed at 25 ± 2 °C for 45 days, the particle size change curve was flat, showing the good storage stability of ASSO-NPs. Nanoparticles need to go through a variety of environments from entering the body to function. Some nanoparticles can be stable in pure water, but may increase in particle size and aggregate sink in the gastrointestinal fluid or blood due to the presence of some proteins or changes in pH [34]. In order to investigate whether the nanoparticles were stable before taking effect and the appropriate way of administration, the stability of ASSO-NPs in normal saline, 5% glucose solution, PBS, artificial gastric juice, artificial intestinal fluid, and plasma was investigated. As shown in Figure 3B, the particle size change curve of ASSO-NPs had almost no fluctuation after incubating in normal saline, 5% glucose solution, and PBS for 8 h. There was a certain fluctuation in the particle size change curve with incubation in artificial gastrointestinal fluid and plasma, but the change in particle size was less than 50 nm, showing the good stability of ASSO-NPs in physiological media. At the same time, it has been proven that ASSO-NPs were suitable for both oral administration and intravenous injection.

### 3.5. The Pharmacological Evaluation of ASSO-NPs

#### 3.5.1. In Vitro Cytotoxicity Assay

**Figure 4:**
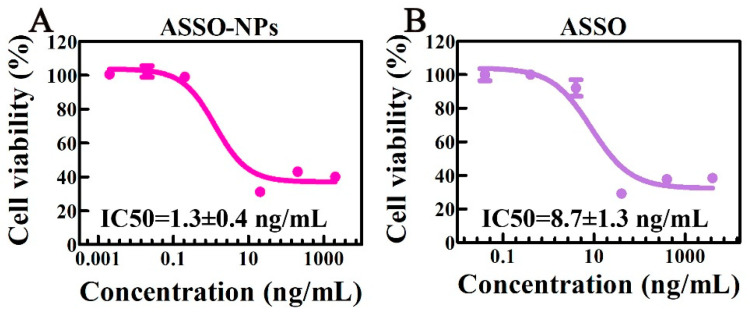
The cytotoxicity of ASSO-NPs or ASSO. Fitting inhibition curve of (**A**) ASSO-NPs or (**B**) ASSO on 4T1 cells.

MTS assay is a commonly used method to detect the cytotoxicity of drugs in vitro [35,36], which has the characteristics of economy and high sensitivity. In the study, 4T1 cells were used to investigate the antitumor activity of ASSO or ASSO-NPs by MTS assay. After being incubated with different concentrations of ASSO or ASSO-NPs for 72 h, 4T1 cells showed different survival rates. According to the change trend of cell survival rate with different concentrations of ASSO or ASSO-NPs, the fitting inhibition curve was obtained, and IC50 was calculated by the software described above (Figure 4), which revealed the strong antitumor activity of ASSO and ASSO-NPs against 4T1 cells. In particular, the IC50 of ASSO-NPs for 4T1 cells was 1.3 ± 0.4 ng/mL, which decreased by 6-fold more than that of ASSO (8.7 ± 1.3 ng/mL), indicating that the cytotoxicity of ASSO-NPs was significantly improved (*p* < 0.05).

Fatty acids are the raw materials for cells to synthesize cell membranes and also play an important role in signal transduction and hormone synthesis [37,38]. Tumor cells need a lot of fatty acids due to rapid division and proliferation. The ASSO was mainly composed of fatty acids [18]. Obviously, tumor cells will take up more ASSO. In addition, free ASSO entered cells mainly through passive transport, while ASSO-NPs can also enter cells through endocytosis [39,40], further enhancing the uptake of ASSO-NPs by tumor cells. This explained that ASSO-NPs showed stronger cytotoxicity than free ASSO.

#### 3.5.2. In Vivo Antitumor Activity in 4T1 Tumor-Bearing Mice

**Figure 5:**
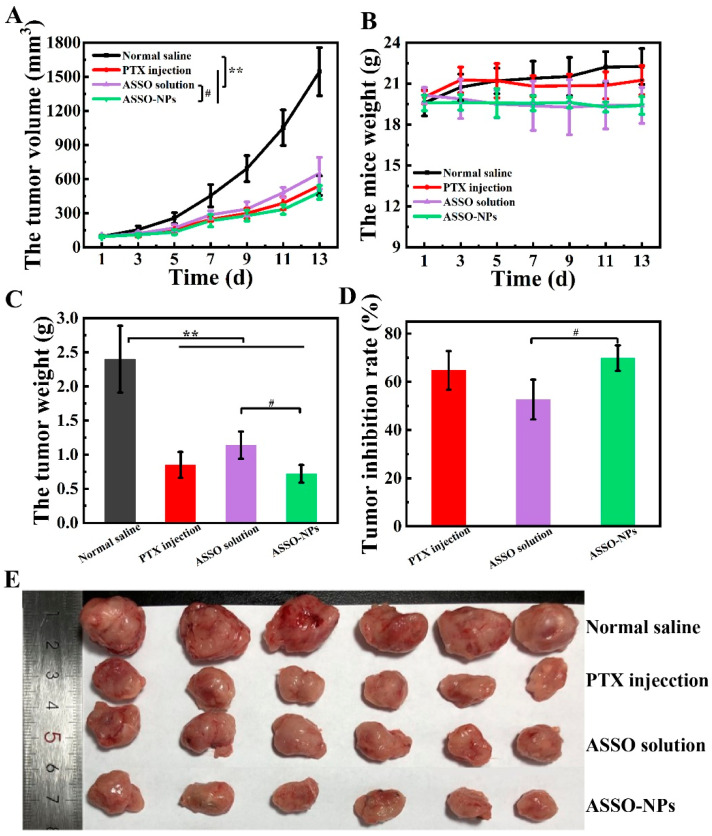
In vivo antitumor activity in 4T1 tumor-bearing mice of ASSO-NPs. (**A**) The change curve of the mice tumor volume over time. (**B**) The change curve of the mice weight over time. (**C**) The tumor weight of all groups; ** *p* < 0.01 vs. normal saline; ^#^ *p* < 0.05 vs. ASSO solution. (**D**) TIR of all experimental groups; ^#^ *p* < 0.05 vs. ASSO solution. (**E**) The actual photo of tumors for each group collected at the end of experiment. Data represent mean ± SD (*n* = 6).

4T1 tumor-bearing mice model was used to investigate the antitumor activity of ASSO and ASSO-NPs. As a broad-spectrum anti-cancer drug, PTX injection is mainly used for the treatment of breast cancer, ovarian cancer, and lung cancer, as a positive drug in this study [41,42]. Figure 5A displayed the changes in the mice tumor volume of all groups during the experiment. The negative control group was given normal saline, and the tumor volume increased rapidly, reaching over 1500 mm^3^ at the end of the experiment, while the tumor volume growth of other experimental groups was inhibited and increased slowly. Especially in the ASSO-NPs group, the tumor volume was only about 500 mm^3^ at the end of the experiment, indicating the strong inhibitory effect of ASSO-NPs on breast cancer. Moreover, the tumor volume of ASSO solution group was slightly larger than that of the positive drug group on day 13, demonstrating that the ASSO itself also has antitumor activity, which is consistent with the literature reports [17,18].

At the end of the experiment, the mice were sacrificed by cervical dislocation, and the tumor, liver, and spleen were dissected. The tumors collected in each group are shown in Figure 5E. The average tumor weight of mice in each group was exhibited in Figure 5C. There was a significant difference between the tumor weight of the experimental groups and the negative control group (*p* < 0.01). The TIR was calculated according to the tumor weight and was displayed in Figure 5D. The TIR in the ASSO-NPs group was significantly higher than that of the ASSO solution group (69.8% vs. 52.7%, *p* < 0.05), which was slightly higher than that of the positive control group (64.8%). From the point of view of TIR, the inhibition effect of ASSO-NPs on breast cancer with only one-ninth of the ASSO solution group exceeds that of ASSO solution at 135 mg/kg (*p* < 0.05), indicating that the antitumor activity was enhanced after ASSO was prepared into nanoparticles.

In addition to antitumor activity, the safety of drugs should also be focused on. During the experiment, changes in the body weight of the mice were monitored (Figure 5B). There was no significant change in the weight of the mice in the other groups, indicating that the ASSO-NPs had good safety. In addition, the liver index and spleen index of the mice were calculated based on the weight of the liver or spleen and body weight of the mice in each group. As shown in Table 3, there was no significant difference between the liver index of the experimental group and the negative control group, while the spleen index of the other experimental groups was significantly different from the negative control group (*p* < 0.01) except for ASSO-NPs group. Judging from the weight change of mice and the liver and spleen index of mice, the safety of ASSO-NPs was the highest when administered intravenously.

### 3.6. In Vivo Biodistribution Study Based ASSO-NPs 

**Figure 6:**
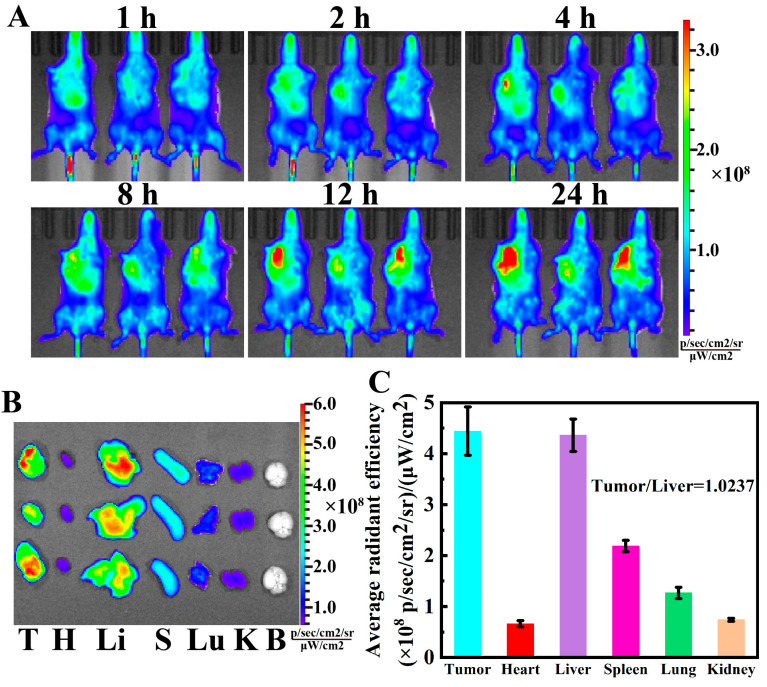
In vivo biodistribution of ASSO-NPs in 4T1 tumor-bearing mice. (**A**) Dynamic biodistribution of ASSO-NPs at different times. (**B**) Biodistribution of ASSO-NPs in isolated organs 24 h after administration, T: tumor, H: heart, Li: liver, S: spleen, Lu: lung, K: kidney, B: brain. (**C**) Fluorescence intensity at different organs was calculated. Data represent mean ± SD (*n* = 3).

Successful delivery of drugs to the tumor site is the key to cancer treatment. In this part, DiR probe was used to label ASSO-NPs to better observe the distribution behavior of ASSO-NPs in tumor-bearing mice. Figure 6A displayed the changes in the distribution of ASSO/DiR-NPs in liver and tumor after entering the body. One hour after administration, the fluorescence intensity of ASSO/DiR-NPs in tumor was similar to that of liver, indicating that ASSO/DiR-NPs could reach the tumor site quickly. From 2 to 24 h after administration, the distribution of ASSO/DiR-NPs in tumor increased gradually and was always obviously more than that in liver. Twenty-four hours after administration, the mice were euthanized and the related organs were taken out for imaging. As shown in Figure 6B, ASSO/DiR-NPs are mainly distributed in the tumor, followed by the liver. The fluorescence intensity of ASSO/DiR-NPs in various organs was calculated, and the results are shown in Figure 6C. The RTTI was 1.0237, demonstrating the excellent tumor delivery ability of ASSO-NPs.

The reason for this phenomenon may be that ASSO contained fatty acids, which are raw materials for the synthesis of cell membranes. In order to meet the needs of rapid proliferation, tumor cells take up large amounts of fatty acids, thus improving the localization of ASSO-NPs at the tumor site. In addition, compared with the nanoparticles prepared by crystalline compounds, ASSO was in the form of liquid, and ASSO-NPs had certain elasticity and flexibility and were prone to deformation, which endowed ASSO-NPs with good permeability [43,44].

**Figure 7:**
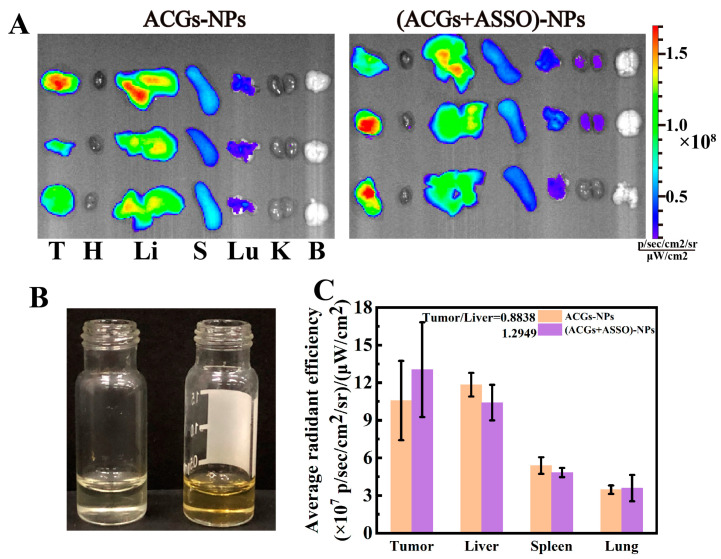
The difference of in vivo biodistribution of ACGs delivered alone and co-delivered with ASSO in 4T1 tumor-bearing mice. (**A**) Biodistribution of ACGs-NPs and (ACGs + ASSO)-NPs in isolated organs 48 h after administration, T: tumor, H: heart, Li: liver, S: spleen, Lu: lung, K: kidney, B: brain. (**B**) Appearance of ASSO without (left) and with ACGs (right). (**C**) Fluorescence intensity in different organs was presented. Data represent mean ± SD (*n* = 3).

In vivo biodistribution data of ASSO-NPs showed that ASSO-NPs had excellent tumor targeting potential. Therefore, ASSO-NPs could be used as a delivery vehicle to deliver chemotherapeutic drugs to the tumor site. Both ACGs and ASSO were extracted from Annona seeds, and ACGs had very good solubility in ASSO (50 mg/mL), which can ensure the basically consistent in vivo behavior of ACGs and ASSO into the body. Figure 7B showed that ACGs was completely dissolved in ASSO, which was clear, but yellower in color than ASSO. ACGs-NPs and (ACGs + ASSO)-NPs containing the same dose of DiR were prepared. Forty-eight hours after intravenous administration, the mice were sacrificed by cervical dislocation, and the tumors and organs were dissected and imaged (Figure 7A). Compared with ACGs-NPs, the fluorescence intensity of (ACGs + ASSO)-NPs in tumor increased by 23% (Figure 7C). The RTTI of (ACGs +ASSO)-NPs was 1.47 times higher than that of ACGs-NPs. These results confirmed that ASSO-NPs can indeed help deliver more ACGs into the tumor.

## 4. Conclusions

In the process of extracting ACGs, ASSO with good antitumor activity can be obtained, but are often discarded and reduce the use value of Annona squamosa. In this study, a very simple method was developed to prepare ASSO-NPs with fewer excipients. ASSO-NPs were uniformly spherical with a small diameter, which had great stability at 25 ± 2 °C for 45 days and in physiological medium. ASSO-NPs displayed stronger antitumor activity than free seed oil against 4T1 cells with the IC50 decreased by about 7-fold. In vivo assay showed that the ASSO-NPs (15 mg/kg) had the highest TIR of 69.8% were and greater than the ASSO solution (52.7%, 135 mg/kg) in 4T1 tumor-bearing mice. Besides, ASSO-NPs had great potential as tumor-targeted delivery vehicles. In vivo distribution data showed that the RTTI of (ACGs and ASSO)-NPs was 1.47-fold that of ACGs delivered alone. 

Delivering more chemotherapy drugs to the tumor site can not only improve its antitumor effect but also reduce the side effects. Currently, the main method to achieve the goal is tumor-targeting delivery. Multi-functional targeted nanoparticles have become common, but require careful design, complex synthesis, and are high cost. In this paper, ASSO-NPs were prepared for the first time with excellent stability and great antitumor activity and tumor delivery efficiency, and are a good prospect in the in vivo delivery of natural bioactive compounds.

## Figures and Tables

**Table 1:** Screening suitable stabilizers of ASSO-NPs (mean ± SD, *n* = 3).

Stabilizer	ASSO/Stabilizer Ratio	Concentration (mg/mL)	DLS Results		
			Size (nm)	PDI	Zeta Potential (mV)
Sodium oleate	2:1	4	321.6 ± 6.3	0.49 ± 0.01	0.3 ± 0.1
Solutol^®^ HS 15			343.0 ± 23.0	0.58 ± 0.03	−33.1 ± 0.9
SDS			311.8 ± 3.8	0.40 ± 0.08	−89.8 ± 2.2
TPGS			253.1 ± 6.7	0.28 ± 0.03	−0.6 ± 0.7

**Table 2:** Further optimization of ASSO-NPs prescription (mean ± SD, *n* = 3).

Stabilizer	ASSO/Stabilizer Ratio	Concentration (mg/mL)	DLS Results		
			Size (nm)	PDI	Zeta Potential (mV)
TPGS	2:1	4	253.1 ± 6.7	0.28 ± 0.03	−0.6 ± 0.7
	1:1	4	259.3 ± 3.3	0.20 ± 0.01	−2.4 ± 0.8
	1:2	4	209.8 ± 2.1	0.21 ± 0.01	−0.6 ± 0.3
	1:2	2	193.7 ± 4.1	0.10 ± 0.09	−0.5 ± 0.3
	1:2	6	258.8 ± 2.5	0.27 ± 0.02	−1.6 ± 0.2

**Table 3:** The liver index and spleen index of all groups (mean ± SD, *n* = 6).

Group	Liver Index	Spleen Index
Normal saline	0.06149 ± 0.00573	0.03423 ± 0.00458
PTX group	0.06484 ± 0.00270	0.02370 ± 0.00304 **
ASSO solution	0.06105 ± 0.00604	0.02452 ± 0.00354 **
ASSO-NPs	0.06648 ± 0.00326	0.03150 ± 0.00490

** *p* < 0.01 vs. Normal saline.

## Data Availability

All data relevant to the publication are included.
